# Transcriptomic Profiling of Femoral Veins in Deep Vein Thrombosis in a Porcine Model

**DOI:** 10.3390/cells10071576

**Published:** 2021-06-22

**Authors:** Leszek Gromadziński, Łukasz Paukszto, Agnieszka Skowrońska, Piotr Holak, Michał Smoliński, Elżbieta Łopieńska-Biernat, Ewa Lepiarczyk, Aleksandra Lipka, Jan Paweł Jastrzębski, Marta Majewska

**Affiliations:** 1 Department of Cardiology and Internal Medicine, School of Medicine, Collegium Medicum, University of Warmia and Mazury in Olsztyn, 10-082 Olsztyn, Poland; 2 Department of Plant Physiology, Genetics and Biotechnology, Faculty of Biology and Biotechnology, University of Warmia and Mazury in Olsztyn, 10-719 Olsztyn, Poland; pauk24@gmail.com (Ł.P.); bioinformatyka@gmail.com (J.P.J.); 3 Department of Human Physiology and Pathophysiology, School of Medicine, Collegium Medicum, University of Warmia and Mazury in Olsztyn, 10-082 Olsztyn, Poland; agnieszka.skowronska@uwm.edu.pl (A.S.); ewa.lepiarczyk@uwm.edu.pl (E.L.); 4 Department of Surgery and Radiology with Clinic, Faculty of Veterinary Medicine, University of Warmia and Mazury in Olsztyn, 10-719 Olsztyn, Poland; piotr.holak@uwm.edu.pl; 5 Clinic of Cardiology and Internal Diseases, University Clinical Hospital in Olsztyn, 10-082 Olsztyn, Poland; smolinskim@interia.eu; 6 Department of Biochemistry, Faculty of Biology and Biotechnology, University of Warmia and Mazury in Olsztyn, 10-719 Olsztyn, Poland; ela.lopienska@uwm.edu.pl; 7 Department of Gynecology and Obstetrics, School of Medicine, Collegium Medicum, University of Warmia and Mazury in Olsztyn, 10-561 Olsztyn, Poland; aleksandra.lipka@uwm.edu.pl

**Keywords:** deep vein thrombosis, RNA-seq, gene expression and regulation

## Abstract

Deep vein thrombosis (DVT) is a severe disease affecting the human venous system, accompanied by high morbidity and mortality rates caused by early and late complications. The study aimed at analyzing the changes in the transcriptome of the femoral vein caused by DVT in the porcine model based on the formation of the thrombus in vivo. The study was performed on 11 castrated male pigs: A thrombus was formed in each left femoral vein in six animals; the remaining five served as a control group. Total RNA was isolated from the left femoral veins of the experimental and control animals. High-throughput RNA sequencing was used to analyze the global changes in the transcriptome of veins with induced DVT. Applied multistep bioinformatics revealed 1474 differentially expressed genes (DEGs): 1019 upregulated and 455 downregulated. Functional Gene Ontology annotated 1220 of DEGs into 225 biological processes, 30 molecular functions and 40 cellular components categories. KEGG analysis disclosed TNF, NF-κB and apoptosis pathways’ overexpression in DVT samples. A thorough analysis of the detected DEGs indicated that a dysregulated inflammatory response and disturbed balance between clotting and anti-clotting factors play a crucial role in the process of DVT.

## 1. Introduction

Deep vein thrombosis (DVT) is a prevailing medical condition caused by the formation of thrombus in veins. Most commonly, DVT affects large veins of the lower extremities [1]. This disease is associated with serious early and late complications, which often follow clot formation. A severe and early complication of DVT is a pulmonary embolism (PE) which develops when a thrombus dislodges from the vein and travels in the bloodstream to reach the pulmonary arteries [2,3,4]. There is an approximately 50% chance that patients with untreated DVT will develop symptomatic PE within 3 months. In 5 to 25% of these patients, PE leads to sudden death [5]. The most common late complication of DVT is post-thrombotic syndrome (PTS)—a form of secondary venous insufficiency affecting about 20–50% of patients within two years from DVT development. PTS manifests with multiple symptoms, such as pain, swelling, heaviness, itching, and cramping in the affected limb [6].

The main factors contributing to DVT include venous stasis, vascular injury and blood hypercoagulability; consequently, the simultaneous incidence of all these three conditions greatly increases the risk of thrombosis. For this reason, the prevalence of DVT is higher during and after surgery, trauma, long-term immobilization and pregnancy, as well as in advanced age or in obese individuals and cancer patients [7]. The clot formation in DVT is a complex and dynamic process, primarily associated with a disturbed balance in the factors controlling the coagulation/fibrinolysis cascades [8]. The clinical studies have suggested that the plasminogen activator inhibitor-1 (PAI-1) plays an essential role in the thrombus creation [9]. Moreover, recent data indicate that activation of immune cells and inflammatory processes are tightly linked with DVT initiation [10]. For example, it has been proven that neutrophil extracellular traps which form in the circulation provide a scaffold that promotes DVT [11]. In a rat model, DVT has been associated with enhanced expression levels of inflammatory factors and a disturbed balance between clotting and anti-clotting mechanisms (i.e., elevated expression and activities of the thrombin-activatable fibrinolysis inhibitor—TAFI—and PAI-1 in vein endothelial cells) [12].

The present research aimed at studying the gene expression profiles associated with DVT. Transcriptome profiling based on the data from high-throughput methods, such as RNA sequencing (RNA-seq), allows us not only to investigate the genetic variation but also mainly focuses on the features and altered expression of genes [13]. It is well known that changes in a single gene and regulatory elements’ expression affect numerous molecular pathways and modulate their function [14]. Thus, the alterations accompanying DVT identified through transcriptome analysis undoubtedly indicate the potential molecular basis of this condition. Taking the above considerations into account, we decided to implement the available bioinformatic tools and resources in our study. This approach should be considered an important step in the comprehensive understanding of the molecular basis of DVT pathogenesis. Due to the logistical difficulty of investigating DVT in humans, several experimental animal models of DVT have been used extensively [15,16,17]. Thus far, transcriptome profiling studies concerning DVT have been investigated in mouse models [18]. However, we decided to perform the thorough bioinformatic analysis to discover the specific molecular pathways that underline the DVT development in the porcine model, due to the similarities in the functional structure of coagulation proteins between humans and pigs [19]. At the deep molecular level, the discovery of specific dysregulated genes may shed new light on how the disturbed interrelationship between endothelium, leukocytes and platelets initiates the DVT inflammatory response. Profound knowledge about the exact mechanisms underlying DVT pathomechanisms is essential in both understanding how to prevent thrombus formation and how to treat this condition.

## 2. Materials and Methods

### 2.1. Surgical Procedures in the Experimental and Control Animals

The study was performed on 11 castrated male pigs (24 weeks old, 60 kg body weight, b.w.) of the Polish Landrace breed; 6 animals served as the experimental group (DVT pigs); the remaining 5 pigs served as controls (CTR). In the experimental group DVT was developed using the porcine model described previously [17]. Briefly, before performing any surgical procedures, all the DVT pigs were pretreated with atropine (Atropinum Sulfuricum, Polfa, Warsaw, Poland, 0.05 mg/kg b.w., s.c.) and azaperone (Stresnil, Janssen Pharmaceutica, Beerse, Belgium, 2.5 mg/kg b.w., i.m.). Thirty minutes later, to induce anesthesia, the main anesthetic drug propofol (Propofol-Lipuro, B. Braun Melsungen AG, Melsungen, Germany, 10 mg/kg b.w.) and the main analgesic drug ketamine (Bioketan, Vetoquinol, Poland, 10 mg/kg b.w.) were given intravenously in a slow, fractionated infusion. The depth of anesthesia was monitored by testing the corneal reflex. Once the animals were transported to an operating theater, general anesthesia during the entire procedure was maintained with inhalation of sevoflurane (Sevoflurane, Baxter, Ontario, CA USA; administered at one human MAC end-tidal concentration of 2.0%) under continuous pulse oximetry and heart-rate monitoring. Under sterile conditions, the left femoral veins were carefully exposed in each animal by dissecting the fascia and exposing and preparing the sartorius muscle. Next, the vein was closed proximally and distally at the length of about 30–40 mm with surgical ligatures. To accelerate the thrombus formation, 200 units of thrombin (BioTrombina, Biomed Lublin S.A., Lublin, Poland) were administered into the closed segment of the vein. Immediately afterwards, to prevent the formation of thrombi outside the closed segments, a bolus of unfractionated heparin (Heparinum WZF, Warsaw, Poland) was administered, in a dose of 100 U/kg, through a catheter inserted in the ear vein. Five hours later (an optimal time needed for thrombus formation after the closing of the femoral vein), all experimental pigs were euthanized with sodium pentobarbital (Euthasol, FATRO, Ozzano dell’Emilia BO, Italy, 140 mg/kg). The left femoral veins were gently exposed and cut open to remove the thrombus with tweezers, and the part of the vein located between the surgical ligatures was collected for transcriptome analyses. The control animals were first pretreated with atropine and azaperone, and thirty minutes later, propofol and ketamine were given intravenously in order to induce anesthesia; next, the control pigs were euthanized with sodium pentobarbital (the drugs, their doses and routes of administration were analogous to those used in the experimental group). Next, the left femoral vein was gently exposed in each control animal and cut out for transcriptome analysis (the samples of the vein were collected in a way which exactly corresponded with that applied in the experimental animals, i.e., both the place of sampling and the length of the vein segment were the same). All the animals were kept under standard laboratory conditions. They were fed standard fodder (Grower Plus, Wipasz, Wadąg, Poland) and had free access to water. The animals were housed and treated according to the guidelines of the local Ethics Committee for Animal Experimentation in Olsztyn (affiliated with the National Ethics Committee for Animal Experimentation, Polish Ministry of Science and Higher Education; decision No. 90/2018 of 13.02.2018). To ensure adequate acclimatization, the pigs were transported from the breeder to the animal quarters 5 days before the scheduled procedure.

### 2.2. RNA Extraction, Library Construction and Sequencing

Within both the experimental and control group, total RNA was isolated from the collected parts of the left femoral veins. RNA was isolated from the left femoral veins taken from all the investigated pigs (*n* = 11). RNA was extracted using a mirVana kit following the manufacturer’s procedure for total RNA purification (Thermo Fischer Scientific, Waltham, MA, USA). The quantity and the quality of the total RNA isolates were evaluated using the Bioanalyzer 2100 (Agilent Technologies, Waldbronn, Germany). Samples were selected for RNA-seq library construction according to the highest RIN values and concentrations. A library was prepared with 1 ug of total RNA for each sample by the Illumina TruSeq mRNA LT Sample Prep kit (Illumina, Inc., San Diego, CA, USA). The first step involved the purification of mRNA molecules using poly-T-attached magnetic beads. Next, the mRNA was cut into small fragments with divalent cations. The cleaved RNA pieces were amplified into the first-strand cDNA using SuperScript II reverse transcriptase (Invitrogen, Waltham, MA, USA) and random primers. In the upstream step, second-strand cDNA synthesis using DNA Polymerase I and RNase H was performed. The purified products of PCR reactions were enriched, and the final cDNAs libraries were constructed. The RNA-seq libraries were quantified using qPCR according to the qPCR Quantification Protocol Guide (KAPA Library Quantification kits for Illumina Sequencing platforms) and qualified using the TapeStation D1000 ScreenTape (Agilent Technologies, Waldbronn, Germany). Indexed libraries were then sequenced using the NovaSeq6000 platform (Illumina, San Diego, CA, USA). The RNA-seq data have been submitted (https://www.ebi.ac.uk/ena, accessed on 4 April 2021) to the European Nucleotide Archive under accession no. PRJEB43020.

### 2.3. Transcriptome Assembly and Identification of Novel Transcripts

The sequencing procedure generated 2 × 151 bp stranded paired-end reads that were evaluated with FASTQC software version 0.11.7 [20]. The Illumina adaptors and low-quality reads (PHRED cut-off score < 20) were removed from downstream analysis using Trimmomatic software v. 0.38 [21]. Quality control was based on the following criteria: all sequences were cropped to 120 bp, and a 10 bp frameshift was verified according to the average PHRED score. After checking the quality attributes, the trimmed paired-end reads were aligned to the pig reference genome with ENSEMBL annotation (Sus_scrofa.Sscrofa11.1.99) adopting the STAR mapper. The following ENCODE parameters were used in the mapping procedure: “-outSAMtype BAM, -SortedByCoordinate, -outFilterType BySJout, -outFilterMultimapNmax 20, -alignSJoverhangMin 8, -alignSJDBoverhangMin 1, -outFilterMismatchNmax 999, -alignIntronMin 20, -alignIntronMax 1000000, -alignMatesGapMax 1000000, -quantMode GeneCounts, -readFilesCommand gunzip, -c, -runThreadN 48”. The Binary Alignment Map files were re-evaluated by the StringTie v. 1.3.3 pipeline [22] to annotate the intergenic-expressed regions and uncover their regulatory properties. The integrity of the control (CTR) and DVT RNA-seq libraries was conducted with ggplot2 Bioconductor packages of R software v.3.9 [23]. The differentially expressed genes (DEGs) were retrieved by a compilation of two statistical methods: DESeq2 [24] and ballgown [25]. Only the DEGs, confirmed by both statistical tools (adjusted *p*-value < 0.05 and |log2FC| > 1) were classified as the final consensus results and transferred to the downstream functional annotated analysis. The ontology and pathway enrichment analysis were achieved with gProfileR [26] based on Gene Ontology (GO) [27] and the Kyoto Encyclopedia of Genes and Genomes (KEGG) [28] databases. Differentially expressed results were illustrated in an MA, a Volcano, a heatmap, GOBubble and Circos plots performed with ggplot2, GOplot and circlize Bioconductor R packages. The pathways’ illustrations were drawn by pathview and KEGG.db R packages. The R script used to perform differential expression analysis and visualization was published in the GitHub repository (https://github.com/prodakt/VTE).

### 2.4. Real-Time PCR

The mRNA level of selected genes was determined by Real-Time PCR. The primers for chosen genes were designed using the Primer3Plus software v.4.1.0 [29] based on the sequences deposited in GenBank and listed in Table S1 in the Supplementary Materials. The cDNA was obtained with the use of the Applied Biosystems High-Capacity cDNA Reverse Transcription Kit (Thermo Fischer Scientific, Waltham, MA, USA, cat. No. 4374966) according to the manufacturer’s protocol. The Real-Time PCR was performed with the use of Applied Biosystems PowerUp SYBR Green Master Mix (Thermo Fischer Scientific, Waltham, MA, USA, cat. No. A25780) according to the manufacturer’s protocol. In brief, each reaction contained 5 μL of master mix (2X), forward and reverse primers in amounts of 500 nM of each, 10 ng of cDNA and a proper volume of nuclease-free water to a final volume of 10 μL. The reactions were performed in four replicates on the QuantStudio 3 Real-Time PCR System (Applied Biosystems; Waltham, MA, USA). The expression of each gene was calculated using the comparative Pfaffl method [30], where the expression is presented as the fold change relative to the untreated control, as well as normalized to an endogenous actin beta (ACTB) as a reference gene (GenBank accession number U07786.1; relative quantification RQ = 1). The results were expressed as means ± standard deviations and presented on a logarithmic scale (log10). Statistical analysis was performed using Student’s *t*-test (two-tailed) in Prism 8 software (GraphPad Software Inc., San Diego, CA, USA). *p*-values were considered statistically significant at <0.05 (*), <0.01 (**) and <0.0002 (***).

## 3. Results

### 3.1. Transcriptomic Signatures of DVT

The summary statistics of 11 cDNA libraries (six from experimental and five from control samples of the left femoral veins) are described in Table 1 and Table 2.

**Figure 1:**
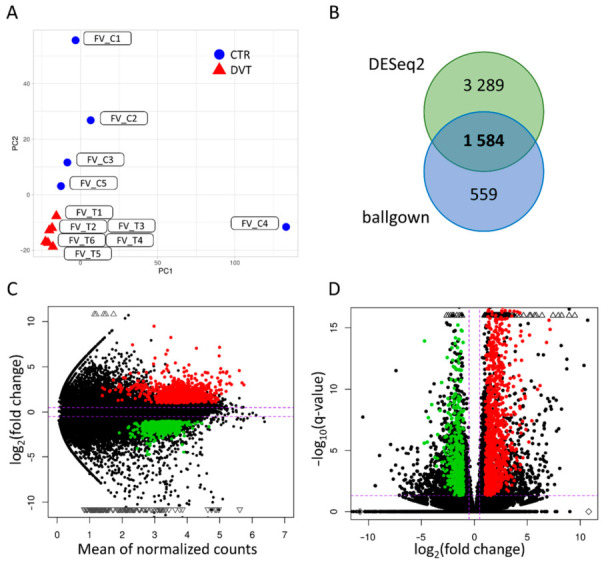
Expression profiles overview of DVT. (**A**) Graphical representation of the first (PC1) and second (PC2) principal components affecting the sample expression pattern of deep vein thrombosis (DVT; *n* = 6) and control (CTR; *n* = 5) libraries. (**B**) Venn diagram with the number of differentially expressed genes (DEGs) tagged by DESeq2 and ballgown methods. The middle number describes the consensus of DEGs distinguished by both methods. (**C**) MA chart with logarithmic values of fold change (logFC; Y axis) plotted against normalized counts (X axis) for DVT-affected and CTR libraries. Two horizontal dotted lines refer to the cut value of log2FC >1 and <−1. (**D**) Volcano plot depicts log2FC plotted against log-normalized *p*-values. The dotted horizontal line indicates negative logarithmic adjusted *p*-value (0.05) cut-off. Dotted vertical lines indicate cut-off values of logFC. (**C**,**D**) Red dots illustrate upregulated differentially expressed genes (DEGs); green dots represent downregulated DEGs; black dots are not significant transcripts, according to DESeq2 and ballgown methods.

After processing, the 373,638,559 clean paired-end reads were uniquely mapped to the Sus_Scrofa.11.1.99 genome, and the mapping results varied from 93.2% to 96.5%. Using the StringTie method, a new annotations file (.gtf) was created and 33,270 transcriptionally active regions (TARs) were assigned. Within obtained annotations, 3628 unknown TARs were identified. Among the 29,642 annotated TARs, 47,125 transcripts were identified as new variants with novel splice junctions. The Euclidean distance divided RNA-seq libraries according to femoral vein thrombosis and control samples (Figure 1A).

### 3.2. Transcript Assembly, Quantification and DE-TARs Analysis

**Figure 2:**
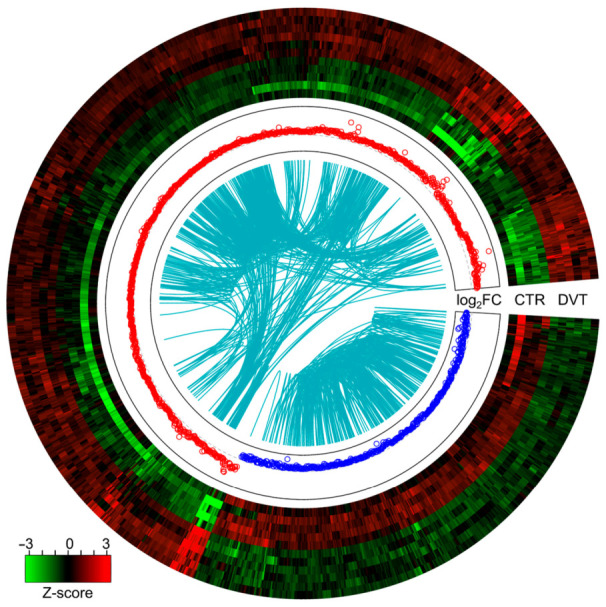
Circular heatmap visualization of differentially expressed genes (DEGs) in the incidence of the DVT. The 11 upper tracks visualize the normalized (Z-score; red-green scale) expression profiles for DEGs in each biological replicate (DVT—deep vein thrombosis and CTR—control libraries). The middle track describes increased (red) and decreased (blue) expression (logFC) in the compared groups. The most-inner track shows the correlation links between the co-expressed DEGs, whereas blue links depict positive Euclidean correlation > 0.9.

In the present RNA-seq study, 1584 differentially expressed TARs (DE-TARs; under the threshold of |log2FC| > 1 and adjusted *p*-value < 0.05) were detected in DVT after the compilation of two statistical methods: DESseq2 and ballgown (Figure 1B–D). Within the TARs, 1474 significant DEGs were identified; of them, 1019 were upregulated and 455 were downregulated (Figure 2; Table S2 in the Supplementary Materials).

**Figure 3:**
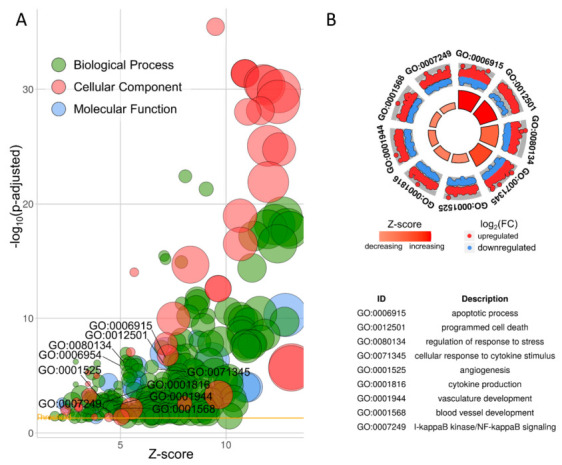
Enrichment ontology visualization. (**A**) GOBubble chart of the ontology terms (biological process—BP, cellular components—CC and metabolic function—MF) detected during Gene Ontology (GO). Circle size is proportional to the logarithmic scale of adjusted *p*-value in enrichment GO analysis. Z-score is calculated from the number of up- and downregulated genes enriched in each GO term. (**B**) Circos visualization of 10 selected BP processes related to DVT. Red dots illustrate upregulated differentially expressed genes (DEGs); blue dots represent downregulated DEGs; black dots are not significant transcripts.

Out of DE-TARs, 1220 genes were assigned to functional Gene Ontology (GO) annotations grouped into 225 biological processes (BP), 30 molecular functions (MF) and the 40 cellular components (CC) categories (Figure 3A,B, Table S3 in the Supplementary Materials).

**Figure 4:**
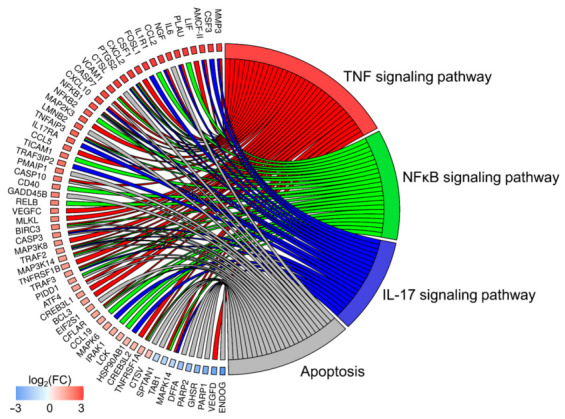
Circos plot represents four significantly enriched Kyoto Encyclopedia of Genes and Genomes (KEGG) pathways associated with differentially expressed genes (DEGs) engaged in deep vein thrombosis (DVT). Gene symbols with logarithmic values (blue-red scale) of fold change (logFC) are located on the left side of circos. Four color links merge genes with KEGG annotations (TNF, NF-κB, IL-17 and apoptosis signaling pathways) on the right side.

The DEGs enriched in these three classes were cross-overlapped. The genes involved in the top five BP class carried out ribonucleoprotein complex biogenesis (90 DEGs out of 361 genes as a count of term size), ribosome biogenesis (73/240), organic substance metabolic process (798/8583), metabolic process (836/9162) and nitrogen compound metabolic process (739/7811; Figure 3a). The top five terms of MF were: binding (946/11,498), RNA binding (127/949), catalytic activity (483/5248), protein binding (601/6853) and transferase activity (222/2102; Figure 3a). In the CC category, the most abundant significantly enriched terms were: nucleolus (144/602), membrane-enclosed, intracellular and organelle lumen (378/2863), intracellular membrane-bounded organelle (753/7516; Figure 3a). To be more specific, we investigated 10 DVT-linked ontology terms connected with angiogenesis, apoptosis, vascularity and immune response (Figure 3b). Meanwhile, the KEGG pathway enrichment analysis revealed that DEGs were categorized into 14 pathways, including the TNF, NF-κB s, IL-17, apoptosis signaling pathways, etc. (Figure 4).

### 3.3. KEGGs Signaling Pathways Analysis

#### 3.3.1. TNF Signaling Pathway

**Figure 5:**
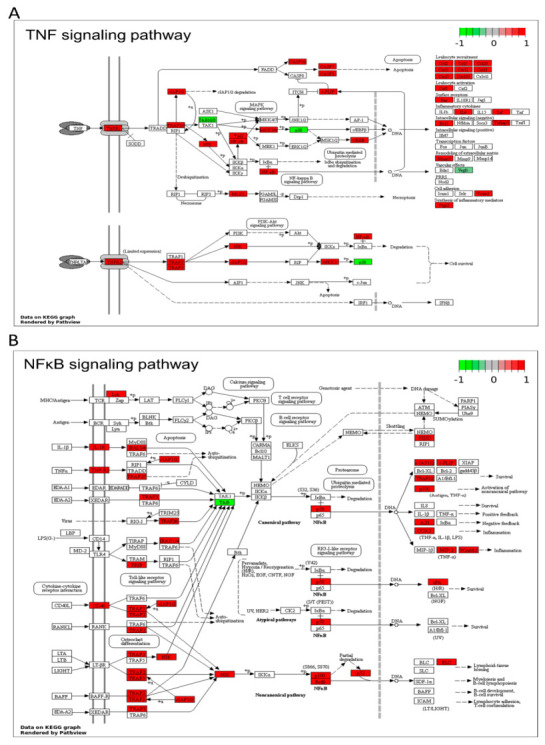
Enrichment Kyoto Encyclopedia of Genes and Genomes (KEGG) analysis of differentially expressed genes (DEGs) engaged in the (**A**) TNF signaling pathway, (**B**) NF-κB signaling pathway. Red and green rectangles present upregulated and downregulated genes, respectively. Logarithmic fold change (logFC; red-green scale) values describe gene expression values.

KEGGs’ signaling pathway analysis disclosed the overexpression of both TNF signaling pathway receptors: TNF Receptor Superfamily Member 1A (TNFR1) and TNF Receptor Superfamily Member 1B (TNFR2) in pigs with induced DVT (Figure 5A). Highly expressed pairs of genes, TNF receptor associated factor 2 (TRAF2) and baculoviral IAP repeat-containing protein 2 (cIAP2 or BIRC3) modulated the TNFR1 signaling complex. Despite inhibition of one of TAK1-TAB components and no significant changes in the inhibitor of the kappa B kinases (IKK) complex, both subunits of NF-κB (encoded by nuclear factor kappa B subunit 1-NF-κB1 and nuclear factor kappa B subunit 2-NF-κB2) indicated upregulation after thrombin formation in the femoral veins. Decreased expression of TGF-beta activated kinase 1 (MAP3K7) binding protein 1 (TAB1) led to downregulation of mitogen-activated protein kinase 14 (p38), the component of p38 mitogen-activated protein kinases complex. A negative effect of TAB1 on p38 was strongly dominant and impaired the upregulation of mitogen-activated protein kinase kinase 3 (MKK3), activator of p38. Downregulation of p38 did not restrain the caspase inhibitor/regulator (CASP8 and FADD like apoptosis regulator-CFLAR) expression. On the contrary, CFLAR was upregulated through the FAS-dependent crosslinking block expression of p38. Overexpression of CFLAR did not influence caspase 8 (CASP8) expression; however, other pro-apoptotic caspases (CASP3, CASP7, CASP10) were inducted in this apoptotic pathway. TNFR1 signaling was modulated by an overexpressed feedback loop composed of CFLAR and cIAP2. Signaling through the TNF pathway aroused the expression of target chemokines (i.e. CCL2, CCL19, CXCL2) and cytokines (IL6, LIF) in DVT samples. The vascular effect was observed in the expression blockage of vascular endothelial growth factor B (VEGFB), vascular endothelial growth factor D (VEGFD) and induction of vascular endothelial growth factor C (VEGFC) during TNF signal transmission.

#### 3.3.2. NF-κB Signaling Pathway

Despite the lack of significant modulation of interleukin 1 Beta (IL-1β) and tumor necrosis factor-Alpha (TNFα) as activators of the canonical nuclear factor-κB (NF-κB) signaling pathway, both receptors (interleukin 1 receptor type 1-IL1R1 and TNFR1) were upregulated in DVT (Figure 5B). However, TAB1 as the main member of NF-κB signaling indicated decreased mRNA expression, and in a consequence inhibited the TAK1-TAB complex, leading to a disturbed canonical NF-κB pathway. Additionally, canonical signaling was modulated by negative feedback controlled by TNF alpha-induced protein 3 (TNFAIP3) overexpression. In DVT, signal transduction through the NF-κB signaling pathway was additionally conducted via an “alternative” pathway, activated by an upregulated CD40 membrane receptor. Overexpression of this molecule triggered the upregulation of TRAF2/3 genes, which were interfered by the cIAP2 inhibitor. The noncanonical transmission was stimulated by the central activator gene-mitogen-activated protein kinase kinase kinase 14 (NIK), which activated NF-κB1 (p50) and NF-κB2 (p52) by binding to TRAF2. DVT promoted the partial degradation of p100 to p52 and sustained formation of the p52/RELB complex in this alternative signaling. The expression levels of the major intracellular members (NF-κΒ2, RELB, NIK and BCL3) of the NF-κB alternative pathway were increased during the thrombus formation. Our research revealed that the downstream effects of NF-κB alternative modulation led to increased activation of the urokinase-type plasminogen activator (PLAU). Moreover, some of the overexpressed target genes were shown to be controlled by NF-κB, therefore affecting inflammation (CXCL2, COX2 and VCAM1) and DNA damage (PIDD).

#### 3.3.3. Apoptosis Signaling Pathway

The apoptosis signaling pathway was investigated to disclose the impact of raised DEGs in DVT (Figure S1 and Table S2 in the Supplementary Materials). Overexpression of the apoptosis initiator caspase—CASP10—and executioner caspases—CASP3 and CASP7—indicated the onset of the programmed cell death. Moreover, an increased expression of the pro-apoptotic and pro-caspase PMAIP1 gene modulated the flexibility of the mitochondrial membrane and efflux of apoptogenic proteins. However, induced expression of resistance genes, TRAF2 and cIAP2, diminished apoptotic susceptibility. Increased activation of the molecular controller (p53-induced death domain protein 1-PIDD) maintained the balance between apoptotic and self-renewal processes upon DNA damage. Other pro-apoptotic factors such as cathepsin L (CTSL) and cathepsin V (CTSV) modulated caspases’ expression through cIAP2. Overexpression of CASP3 and CASP7 led to the downregulation of spectrin alpha, non-erythrocytic 1 (SPTAN1), poly(ADP-ribose) polymerase 1 (PARP1), poly(ADP-ribose) polymerase 2 (PARP2) and DNA fragmentation factor subunit alpha (DFFA) genes, which cooperatively function in the DNA fragmentation, DNA repair failure, cell shrinkage and membrane bubbling.

### 3.4. Validation of the Results

**Figure 6:**
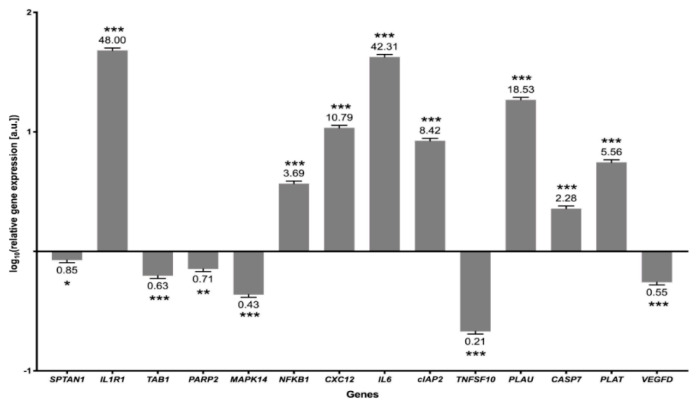
The mRNA expression of selected genes obtained from Real-Time PCR. The values (above and beneath the bars) indicate the changes relative to the normalized expression value of the reference gene ACTB. Arbitrary unit value is set as 1 [a.u.]. Genes with expression value <1 were downregulated and >1 were upregulated. All the relative expression values were presented in logarithmic scale (log10). *p*-values are considered statistically significant at <0.05 (*), <0.01 (**) and <0.0002 (***).

The NGS method was validated using Real-Time PCR which revealed an overexpression of eight genes: IL1R1, NF-κB1, CXCL2, IL6, cIAP2, PLAU, CASP7 and PLAT, whereas underexpression of another six genes: SPTAN1, TAB1, PARP2, MAPK14, TNFSF10 and VEGFD (Figure 6). The highest expression of genes (CXCL2, IL1R1, IL6, PLAU) was noted in the NF-κB and TNF pathways (over 10 arbitrary units compared to the control). Lower, but still significant, expression was noted in the genes of the TNF pathway, where NF-κB1, cIAP2, CASP7 were upregulated in DVT compared to the control libraries. However, TAB1, MAPK14 and VEGFD, which are the TNF signaling markers, were significantly downregulated in DVT libraries. Three of the validated genes, TNFSF10, PARP2 and SPTAN1, engaged in apoptosis signaling were also downregulated (Figure 6).

## 4. Discussion

The present research focused on DVT-associated changes in gene expression profiles in the vein samples. To reach this goal, we performed a high-throughput sequencing which revealed the transcriptome modulation of the DVT. This condition is characterized by the accumulation of platelets, coagulation factors and interactions between the vein wall and inflammatory cells under variable flow conditions [31]. According to the functional enrichment analysis of DEGs, our findings suggest that TNF, NF-κB and apoptosis pathways should be considered in the thrombus formation.

Members of the TNF superfamily can send both survival and death signals to cells [32]. Moreover, TNFα can affect thrombus formation, remodeling and resolution due to the regulation of plasminogen activators’ and inhibitors’ expression [33]. These various effects are transmitted by two receptors: TNFR1 and TNFR2 [34]. Both TNFR1 and TNFR2 signaling complexes converge towards NF-κB activation [35,36]. Nevertheless, TNFα signaling via TNFR1 would be key for the thrombus resolution by improving fibrinolysis, collagenolysis and neovascularization, while TNFα signaling by acting on TNFR2 demonstrates the prothrombotic effects [37]. The KEGGs analysis performed in the present experiment disclosed the upregulation of the TNFR1 and TNFR2, as well as other elements of the TNFα pathway in pigs with induced DVT. On the other hand, this upregulation was accompanied by a decreased expression of the p38, which may be associated with impaired thrombosis and hemostasis, as well as enhanced inflammatory response, ventricular remodeling and cardiac function [38]. The present data revealed that TNFR1 signaling was modulated by an overexpressed feedback loop of CFLAR and cIAP2, and its downstream effect was executed by an increased expression of target chemokines (i.e., CCL2, CCL19, CXCL2), cytokines (IL6, LIF) and vascular endothelial growth factors (VEGFC) in DVT samples. Other members of the VEGF family, such as VEGFB and VEGFD, displayed decreased expression, which might indicate dysregulation of endothelial cell growth and proliferation [39], pericyte recruitment and vascular permeability [40]. The obtained results suggest that the inflammation process plays an overriding role in the formation of DVT. CCL2, expressed by monocytes, macrophages, vascular endothelial cells, smooth muscle cells and fibroblasts, initiates an inflammatory response by inducing monocytes, T cells, eosinophils, basophils and platelets to release other inflammatory mediators [41,42]. We have found that the increased CCL2 activity was accompanied by an upregulation of CCL5, which exerts a pro-angiogenic activity [43]. Moreover, high expression of CXCL2, a pro-inflammatory chemokine that induces neutrophil aggregation and neutrophil extracellular trap formation [44,45], could possibly affect the mechanisms of thrombus formation and resolution. In the analyzed porcine DVT model, the downstream effect of the TNF signaling pathway was also manifested through an increased expression level of the IL6 cytokine, which could lead to enhanced pro-coagulant activity and thus clotting activation [46,47]. In vivo research indicated that a lower IL6 level correlated with decreased expression of chemokines and adhesion molecules and thus reduced the thrombus size [48]. Therefore, the results of the present experiment indicate that neutralization of IL6 expression might be one of the major therapeutic challenges in VTE patients.

The present results indicate that another signaling pathway with significantly overexpressed genes in DVT pigs was NF-κB. This pathway plays an important role in the activation of platelets during thrombosis and inflammation [49,50], as its activation is correlated with molecular mechanisms involved in the pathophysiology of DVT [51]. Depending on the receptor triggering intracellular signaling, the activation of the NF-κB pathway may be canonical or noncanonical [52]. We detected an increased expression of the IL1R1 and CD40 molecule, initiating the canonical and noncanonical NF-κB activation, respectively. Moreover, our results showed an upregulation of genes associated with these two distinctive pathways, including p50/RELA and p52/RELB. However, the following downstream of the main signaling components of NF-κB activation may suggest a predominance of the noncanonical pathway in veins with induced DVT. For example, we have observed an overexpression of the TRAF2 in the noncanonical pathway that ultimately leads to phosphorylation, ubiquitination and processing of p100 into the active subunit p52 [53]. Our data confirm the upregulation of further major intracellular members (NF-κΒ2, RELB, NIK and BCL3) of the NF-κB alternative pathway that is associated with thrombo-inflammatory processes [50]. However, the negative regulation of the canonical pathway may be the premise pointing to noncanonical NF-κB pathway activation as a background for the DVT. For instance, increased expression of TAK1 and TAB1 strongly induces NF-κB activation [54], and we found that in DVT samples, the TAB1 mRNA level was decreased, which may further inhibit the TAK1-TAB complex and probably disturb the canonical NF-κB pathway. Additionally, our results revealed increased transcription activity of TNFAIP3 that may be involved in negative feedback modulation of the NF-κB canonical signaling [55]. However, considering DVT etiology, it should be emphasized that stimulation of the noncanonical signaling can also activate components of the classical pathway and that the transcriptional responses of both pathways can be qualitatively very similar [56].

Except for implication of NF-κB in maintaining pro-survival and pro-inflammatory states in vascular bed and blood cells [49], this complex can also induce a variety of coagulation factors, including tissue factor (TF), factor VIII, PLAU and plasminogen activator inhibitor-1 (PAI-1) [50]. Our results revealed that with respect to DVT samples, the upregulation of p50 led to the overexpression of both activators (PLAU) and inhibitors (PAI-1) of the blood coagulation pathway. It has been found that inhibition of p50 is conducive to partial suppression of DVT [57], and it corresponds to decreased levels of PAI-1, TNF-α, IL-6, IL-8 in a rat model [48]. These findings are in line with clinical research which suggests that the activity of PAI-1 plays an important role in the initiation and development of DVT [58]. Moreover, it has been found that thrombin generation and fibrin formation may also be modulated by TF and tissue factor pathway inhibitors (TFPI) [59]. TF overexpression observed in experimental pigs may again be connected with increased expression of transcription factor p50, as it has been revealed that p50 plays a crucial role in the transcriptional regulation of TF in vascular endothelial cells and monocytes/macrophages activated in DVT pathogenesis [57]. Additionally, our experiment revealed increased expression of TFPI and selectin P (SELP), established as diagnostic biomarkers in acute DVT [60,61]. Detected genes that were differentially expressed confirmed that stimulation of the NF-κB signaling and a disturbed balance between clotting and anti-clotting factors resulted in the thrombus rise [12].

Furthermore, in the DVT samples, we discovered increased transcriptional activity of prostaglandin-endoperoxide synthase 2 (COX2), which may be a downstream effect of the TNF and NF-κB pathways’ modulation. In physiological conditions, COX2 expression is absent in most tissues but is induced by proinflammatory and proliferative agents after exposure to harmful stimuli [62]. The COX2 expression is important in protecting heart cells damaged by ischemia and is a sensitive indicator of vessel damage [63]. Moreover, it was reported that COX2 may have a direct effect on atherogenesis [64]. The increased expression of the vascular cell adhesion molecule 1 (VCAM1) detected in the present study may also be an effect of the stimulation of both the TNF and NF-κB pathways [65,66]. The upregulation of the local expression of VCAM1 is commonly observed in chronic venous insufficiency and venous hypertension [67]. Moreover, it has been found that patients with DVT had a much higher concentration of circulating VCAM1 compared to patients without thrombosis [68]. Expression of VCAM1 may also be induced by IL-17A upregulation in endothelial cells [69]. Although the present data did not reveal IL-17A gene differentiation, the increased transcriptional activity of the IL-17A gene receptor (IL17RA) was observed. This, in turn, could lead to an increased cell sensitivity to IL-17A and therefore to activation of the IL17 signaling pathway and enhanced production of CXCL2, CXCL10 or IL6, etc. This phenomenon may play a crucial role in thrombus formation, as simultaneous activation of TNF and IL-17 pathways in endothelial vein cells favors platelet aggregation and the initiation of neutrophil extracellular traps [69,70,71].

Moreover, the KEGG analysis revealed that an increased expression of TNF receptors, besides regulating TNF and NF-κB pathways, probably also affected apoptosis in the DVT. This finding is consistent with the previous suggestion that in the pathogenesis of unprovoked VTE, overexpression of TNFRs increases the apoptosis rate and decreases proliferation and endothelial repair, leading to increased thrombus formation. Theoretically, the detected downregulation of p38 observed in DVT samples should significantly decrease platelet apoptosis [72]. However, despite the downregulation of p38, its inhibiting impact was probably diminished by an overexpression of apoptosis initiator caspase—CASP10—and executioner caspases—CASP3 and CASP7—which indicates the onset of programmed cell death. Additionally, we identified an increased expression of pro-apoptotic sensitizer/de-repressor—PMAIP1—which neutralizes anti-apoptotic agents and promotes efflux of apoptogenic proteins [73].

## 5. Conclusions

In conclusion, we performed a thorough bioinformatic analysis of the femoral vein samples taken from control animals and animals with induced DVT, which revealed that dysregulation of TNF, NF-κB and apoptosis pathways affects inflammatory responses as well as the formation, remodeling and resolution of the thrombus in DVT pathogenesis. These findings may be profound in comprehending the intricate interrelationship between endothelium, leukocytes and platelets during the inflammatory response, which, if disturbed, may ultimately lead to DVT. Further investigations of the DVT molecular background should involve proteomic follow up of the currently identified DEGs. Performed identification of the genes dysregulated in the transcriptomic level may set the direction for future in vitro flow-based research. Moreover, we could hypothesize that using untargeted metabolomics as a discovery platform, coupled with functional studies, could serve as a relatively unbiased approach for revealing new metabolic pathways in the pig’s model. This could contribute to the understanding of DVT pathogenesis and its adverse complications. Therefore, we hope that our research will lay the groundwork for future mechanistic studies, clinical trials and new therapeutic agents.

## Figures and Tables

**Table 1:** The statistical metrics for the RNA libraries.

RNA-Seq Libraries	Number of Raw Reads	Number of Processed Reads	Number of Uniquely Mapped Reads	Uniquely Mapped Reads (%)	Number of Multimapped Reads
FV_C1	31,683,264	29,014,357	27,462,945	94.65	764,528
FV_C2	36,724,405	33,348,453	31,094,279	93.24	1,740,840
FV_C3	39,861,429	36,179,831	34,714,702	95.95	850,949
FV_C4	40,514,407	35,976,149	34,248,079	95.20	928,229
FV_C5	32,653,642	29,745,640	28,547,359	95.97	617,930
FV_T1	41,965,137	38,446,156	36,212,924	94.19	868,794
FV_T2	41,704,895	38,432,218	36,876,819	95.95	926,073
FV_T3	32,831,286	29,868,416	28,793,908	96.40	590,702
FV_T4	38,083,555	34,833,923	33,551,704	96.32	756,735
FV_T5	41,165,295	37,191,583	35,210,456	94.67	1,236,692
FV_T6	33,899,526	30,601,833	29,534,470	96.51	637,436

FV_C refers to femoral vein controls; FV_T refers to experimental samples. “Raw reads” refer to reads obtained from sequencing protocol; “Processed reads” refer to reads that passed the quality control procedure; “Uniquely mapped reads” refer to reads that were mapped to a unique (only one) location of the reference genome; “Multimapped reads” refer to reads aligned to more than one locus on the reference genome.

**Table 2:** Percentage of paired-end reads mapped to the genome. Reads were aligned according to the genomic localization; such a coding region represented by exons, untranslated region (UTR) represented by 5′ and 3′ UTR, introns and intergenic region located between the genes.

RNA-Seq Libraries	Reads Mapped on Codding Regions (%)	Reads Mapped on UTR Regions (%)	Reads Mapped on Intronic Regions (%)	Reads Mapped on Intergenic Regions (%)
FV_C1	45.30	28.31	10.91	15.47
FV_C2	46.08	27.59	11.65	14.68
FV_C3	47.10	29.98	9.05	13.87
FV_C4	65.44	16.07	6.65	11.84
FV_C5	46.41	28.41	10.18	15.00
FV_T1	45.11	29.44	11.02	14.42
FV_T2	45.78	30.39	10.21	13.62
FV_T3	44.20	30.73	10.16	14.91
FV_T4	42.98	30.79	11.94	14.29
FV_T5	47.00	30.92	8.48	13.60
FV_T6	46.11	32.17	8.39	13.33

FV_C refers to femoral vein controls; FV_T refers to experimental samples.

## Data Availability

The RNA-seq data underlying this article have been submitted (https://www.ebi.ac.uk/ena) to the European Nucleotide Archive under accession no. PRJEB43020.

## Supplementary Materials

The following are available online at https://www.mdpi.com/article/10.3390/cells10071576/s1, Figure S1: Enrichment Kyoto Encyclopaedia of Genes and Genomes (KEGG) analysis of differentially expressed genes (DEGs) engaged in the apoptosis signalling pathway.

## Abbreviations

VTE	venous thromboembolism
DVT	deep vein thrombosis
PE	pulmonary embolism
DEGs	differentially expressed genes
GO	Gene Ontology
KEGG	Kyoto Encyclopedia of Genes and Genomes
RNA-Seq	RNA Sequencing
CTPA	computed tomography pulmonary angiography
CTR	control
TARs	transcriptionally active regions
DE-TAR	differentially expressed TARs
BP	biological processes
MF	molecular functions
CC	cellular components
TNFR1 (TNFRSF1A)	TNF Receptor Superfamily Member 1A
TNFR2 (TNFRSF1B)	TNF Receptor Superfamily Member 1B
TRAF2	TNF receptor associated factor 2
TRAF3	TNF receptor associated factor 3
cIAP2 (BIRC3)	baculoviral IAP repeat-containing protein 2
TAK1 (MAP3K7)	mitogen-activated protein kinase kinase kinase 7
TAB1 (MAP3K7IP1)	TGF-beta activated kinase 1 (MAP3K7) binding protein 1
NF-κB1(p50)	nuclear factor kappa B subunit 1
NF-κB2 (p100; p52)	nuclear factor kappa B subunit 2
MAPK14 (p38)	mitogen-activated protein kinase 14
MKK3 (MAP2K3)	mitogen-activated protein kinase kinase 3
CFLAR	CASP8 and FADD like apoptosis regulator
CASP	caspase; CCL2: chemokine (C-C motif) ligand 2
CCL5	C-C motif chemokine ligand 5
CCL19	C-C motif chemokine ligand 19
CXCL2	chemokine (C-X-C motif) ligand 2
CXCL10	C-X-C motif chemokine ligand 10
IL6	interleukin 6
LIF	interleukin 6 family cytokine
VEGFB	vascular endothelial growth factor B
VEGFD	vascular endothelial growth factor D
VEGFC	vascular endothelial growth factor C
IL-1β	interleukin 1 Beta
IL1R1	interleukin 1 receptor type 1
TNF	tumor necrosis factor
TNFα	tumor necrosis factor-Alpha
TNFAIP3	TNF alpha induced protein 3
CD40	CD40 molecule
NIK (MAP3K14)	mitogen-activated protein kinase kinase kinase 14
RELB	RELB proto-oncogene, NF-kB subunit
RELA	RELA proto-oncogene, NF-kB subunit
BCL3	BCL3 transcription coactivator
PLAU	plasminogen activator, urokinase
PLAT	plasminogen activator, tissue type
COX2 (PTGS2)	prostaglandin-endoperoxide synthase 2
VCAM1	vascular cell adhesion molecule 1
PIDD	p53-induced death domain protein 1
PMAIP1 (NOXA)	phorbol-12-myristate-13-acetate-induced protein 1
CTSL	cathepsin L
CTSV	cathepsin V
SPTAN1	spectrin alpha, non-erythrocytic 1
PARP1	poly(ADP-ribose) polymerase 1
PARP2	poly(ADP-ribose) polymerase 2
DFFA	DNA fragmentation factor subunit alpha
TNFSF10 (TRAIL)	TNF superfamily member 10
TF	tissue factor
PAI-1 (Serpine 1)	plasminogen activator inhibitor-1
TFPI	tissue factor pathway inhibitor
SELP	selectin P
IL-17A	interleukin 17A
IL17RA	interleukin 17 receptor A
