# Supplementary material for: Transcriptomic Profiling of Femoral Veins in Deep Vein Thrombosis in a Porcine Model
Source: Cells. 2021 Jun 22;10(7):1576. doi: 10.3390/cells10071576 (PMC8304794; doi:10.3390/cells10071576)
Supplement: Supplementary file 1 [file cells-10-01576-s001.zip › cells-1233607-supplementary materials.pdf]

Supplementary Materials

Transcriptomic Profiling of Femoral Veins in Deep Vein Thrombosis in a Porcine Model

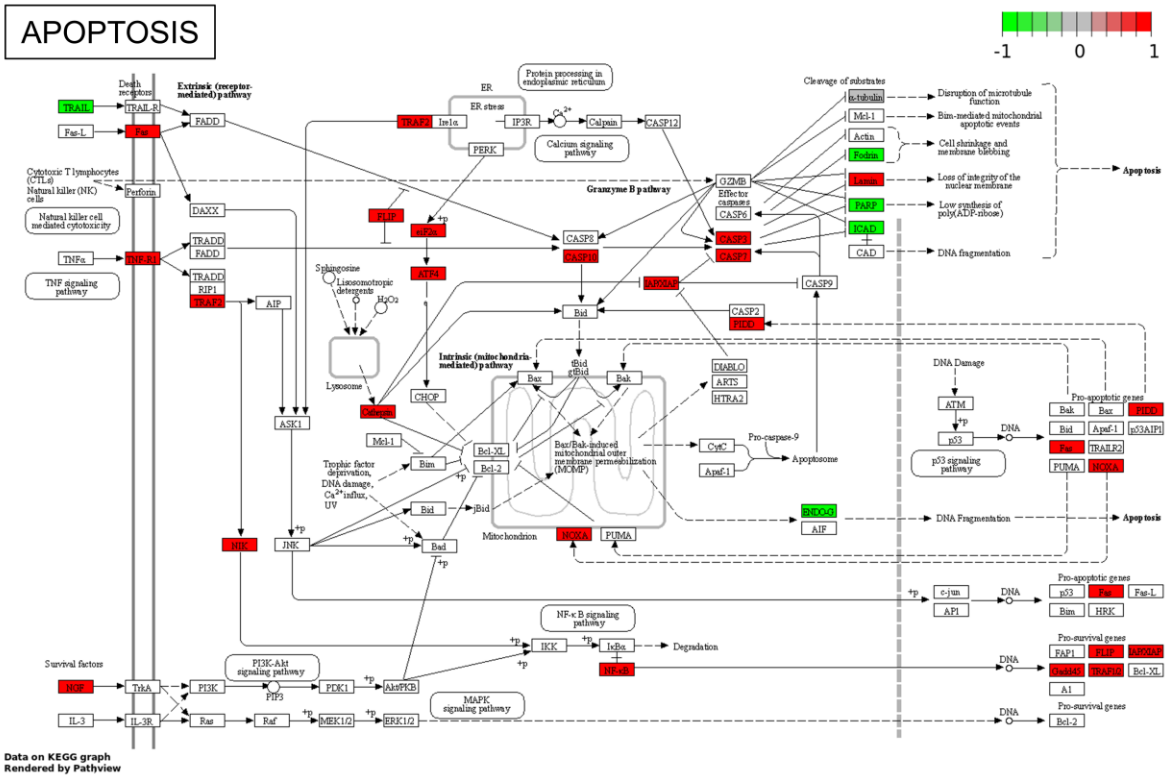

Figure S1. Enrichment Kyoto Encyclopaedia of Genes and Genomes (KEGG) analysis of differentially expressed genes (DEGs) engaged in the apoptosis signalling pathway. Red and green rectangles present upregulated and downregulated genes, respectively. Logarithmic fold change (logFC; red-green scale) values describe gene expression values.

Table 1. The list of primers used for Real-time PCR.

Name	Forward sequence: (5' to 3')	Reverse sequence: (5' to 3')
SPTAN1	CAGCAGTGGATCAACGAGAA	CGCCACCTTGTTAATGTCCT
IL1R1	AGACTACCGGTTGCAGGAGA	CGGTGGTCACATTTGCTATG
TAB1	TCCTGTACGGGGTCTTCAAC	ACTCCAGGAAGCTCCTCTCC
PARP2	AACTGGGTGGGAATCCTGAG	TGGTGCTATGCTTGTCCTGA
MAPK14	GGGGCAGATCTGAACAACAT	GGCCACATAGCCTGTCATTT
NFKB1	AGCTCTCCTCAAAGCAGCAG	TGCTTCATGTCTCCTTGTGC
CXC12	CTGCTGCTCCTGCTCCTAGT	TGACTTCCGTTTGGTCACAG
IL6	CACCAGGAACGAAAGAGAGC	GTTTTGTCCGGAGAGGTGAA
cIAP2	AGCTTGCAAGTGCAGGTTTT	GCTTGAATGCGACTGATGAA
TNFSF10	CAACAAGGCATTCCTCACCT	CCAGCTCTCCATTCCTCAAG
PLAU	TCACCACCAAAATGCTGTGT	CTCTCCCCCAACATGAGTGT
CASP7	TCAAGGAGCTGTCCTGGAGT	GTGCCTGAATCAGCAGTGAA
PLAT	TGGAGCACAACAGAGAGTGG	TGCAGAAATCGGGGCTATAC
VEGFD	TCCAGGAACTGGCTCTCTGT	CCGTTCGTACAGGTTCTGGT
ACTB	GGATGCAGAAGGAGATCACG	ATCTGCTGGAAGGTGGACAG

Table 2. Differentially expressed protein-coding genes (DEGs) identified in the femoral veins with induced deep vein thrombosis.

Table 3. Gene Ontology enrichment classification of protein-coding genes (DEGs). adjusted p-value with cut-off < 0.05; BP—biological process; MF—molecular function; CC—cellular components; Term size - number of genes annotated in GO database terms; Query size - number of all DEGs assignment to GO category; Intersection size - number of the DEGs assignment to particular GO term.
